# Enforcing Ising-like magnetic anisotropy *via* trigonal distortion in the design of a W(v)–Co(ii) cyanide single-chain magnet†
†This project was granted financial support from the Department of Energy (DE-FG02-02ER45999). Funds for the SQUID magnetometer were obtained from the National Science Foundation.
‡
‡Electronic supplementary information (ESI) available. CCDC 1508991. For ESI and crystallographic data in CIF or other electronic format see DOI: 10.1039/c7sc02925j


**DOI:** 10.1039/c7sc02925j

**Published:** 2017-11-13

**Authors:** Yuan-Zhu Zhang, Brian S. Dolinar, Shihao Liu, Andrew J. Brown, Xuan Zhang, Zhao-Xi Wang, Kim R. Dunbar

**Affiliations:** a Department of Chemistry , Texas A & M University , College Station , TX 77842 , USA . Email: dunbar@chem.tamu.edu ; Fax: +1 979 845 7177; b Department of Chemistry , Southern University of Science and Technology , Shenzhen , 518055 , P. R. China

## Abstract

A new octacyanotungstate(v) based single chain magnet {[(Tpm)Co(DMF)W(CN)_8_]_2_[Co(DMF)_4_]·2DMF}_*n*_ (**1**, Tpm = 1,1,1-trispyrazoylmethane), with an effective barrier of 39.7(3) cm^–1^ is reported. The Ising-like magnetic anisotropy of the chain originates from the nearly parallel local orientations of the Co(ii) ions with the source of the uniaxial magnetic anisotropy being a trigonal distortion of the octahedral environment with the *fac*-tridentate capping Tpm ligand.

## Abstract 2

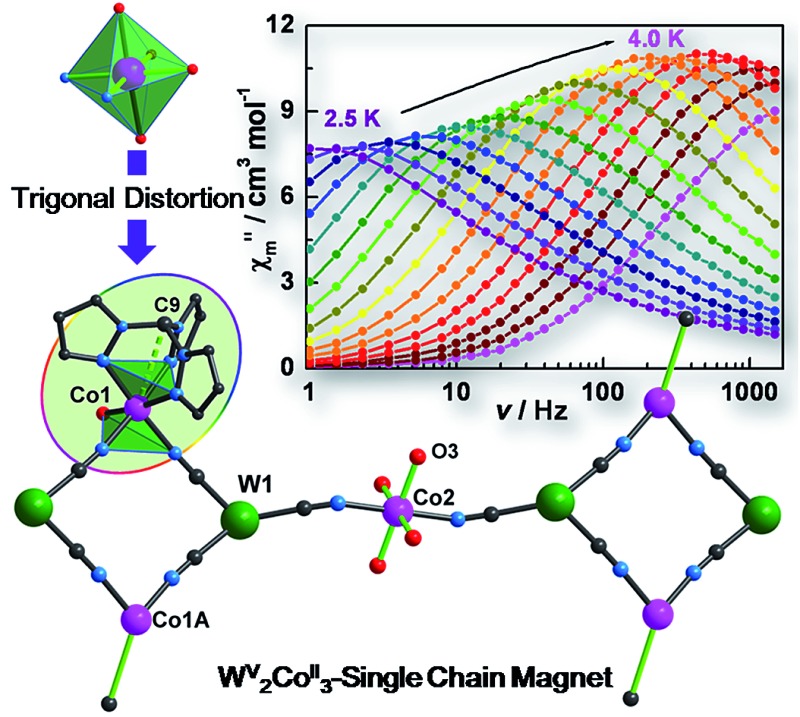
A new octacyanotungstate(v) singe chain magnet with an effective energy barrier of 39.7(3) cm^–1^ is achieved by enforcing Ising-like magnetic anisotropy *via* introduction of trigonal distortion with a *fac*-tridentate capping ligand.

## Introduction

Since the first experimental verification of Glauber dynamics^1^ for a cobalt(ii)–nitronyl nitroxide radical chain complex in 2001,^2^ the phenomenon of slow relaxation in 1-D Ising paramagnets, known as single-chain magnets (SCMs),^3^ has received a great deal of attention in the field of molecular magnetism.^4^ The overall energy barrier (Δ_τ_) for reversing the direction of magnetization of an SCM is a sum of the correlation (Δ_ξ_) and anisotropy (Δ_A_) energies. As such, SCMs have the potential to exhibit higher blocking temperatures than their zero-dimensional counterpart, namely single-molecule magnets (SMMs) because of the additional correlation energy and the potential to optimize the magnetic coupling (*J*) and zero field splitting (*D*). For example, the introduction of bulky, rigid pyrene into the Co(ii)–nitronyl nitroxide radical system led to stronger magnetic coupling, which in turn resulted in a SCM with a record blocking temperature of 14 K.^5^

An increasing number of SCMs are being realized from the application of design principles involving the selection of specific bridging units in combination with polynuclear anisotropic metal complexes with ancillary blocking ligands.^6^ The use of polynuclear SMMs as building blocks for the construction of SCMs represents a promising strategy, but enforcing the proper alignment of the subunits to afford Ising-like anisotropy is a challenge.^7^ In this respect, the use of mononuclear SMMs as building blocks is an excellent approach to SCM design, as a number of these molecules have been found to exhibit high energy barriers due to the presence of large uniaxial anisotropy stemming from spin–orbit coupling and specific geometries and they possess only one easy axis to control.^8^ The use of an efficient bridging ligand is of utmost importance in the design of SCMs. In this vein, cyanometallate complexes have received considerable attention because of the efficient exchange coupling through the cyanide bridge and the prospect for preparing homologous series of magnetic materials using a modular approach.^9^ Modifications of cyanometallate complexes are readily accomplished *via* installation of suitable capping ligands which allow for effective tuning of the ligand field, redox potentials, and electronic configurations of the metal centers. Such adjustments had led to the realization of new functional materials including SMMs,^10^ SCMs^11^ and photomagnets.^12^

In the field of cyanometallate molecular magnets, a common building block that has been widely studied is the octacyanotungstate(v) ion which, as a 5d transition metal complex,^13^ exhibits an enhanced ability for magnetic coupling through bridging ligands due to its diffuse orbitals. The introduction of Mn(iii) Schiff base units as a source of anisotropy into compounds of [W(CN)_8_]^3–^ resulted in the realization of SCMs,^14^ but there are few other examples of chain compounds that behave as SCMs.^15^ In 2003, Li *et al.* reported a 3,2-chain compound {[Co(DMF)_4_W(CN)_8_]_2_[Co(DMF)_4_]}_*n*_ (1*), which exhibits long range magnetic ordering below 8 K with coexistence of spin-glass behaviour.^16^ Later, Sieklucka and co-workers as well as the Dunbar group worked on modifying the Co(ii) sites with *mer*-tridentate planar ligands which resulted in the isolation of the chain compounds ({[Co(iPr-Pybox)(MeOH)]_3_[W(CN)_8_]_2_·5.5MeOH·0.5H_2_O and [(tptz)Co(H_2_O)W(CN)_8_]_2_[Co(H_2_O)_4_]·2H_2_O}_*n*_ (iPr-Pybox = 2,2′-(2,6-pyridinediyl)bis(4-isopropyl-2-oxazoline); tptz = 2,4,6-tris(2-pyridyl)-1,3,5-triazine).^17^ Both compounds exhibit the onset of frequency dependent out-of-phase signals in the AC susceptibility data at ∼2 K but these results do not conform to *bona fide* SCM behavior. Nevertheless the findings hint at the potential for tuning the magnetic behaviour of such systems by modifying the coordination environment of the Co centers with different capping ligands in order to increase the uniaxial magnetic anisotropy.

Recently, we discovered that axial trigonal distortion of the crystal field affords a huge uniaxial magnetic anisotropy with a *D* value on the order of –100 cm^–1^ for the mononuclear cobalt(ii) SMM [Co(Tpm)_2_](BPh_4_)_2_ (Tpm = 1,1,1-trispyrazol methane).^18^ Taking a cue from this work and the preceding discussion, the premise behind the present work was to incorporate these highly anisotropic, trigonally distorted cobalt SMMs into cyanometallate-based chain structures in order to probe if enhanced SCM behavior could be engendered. Herein we report a chain compound with a 3,2-chain structural archetype, *viz.*, {[(Tpm)Co(DMF)W(CN)_8_]_2_[Co(DMF)_4_]}_*n*_·2*n*DMF (**1**).§
§Crystal data for **1** (CCDC 1508991): C_60_H_76_Co_3_N_36_O_8_W_2_, F.W. = 1974.06, triclinic, space group *P*1, *a* = 11.910(3), *b* = 12.221(3), *c* = 15.587(4) Å, *α* = 69.947(3)°, *β* = 81.802(3)°, *γ* = 66.513(3)°, *V* = 1954.5(9) Å^3^, *T* = 110(2) K, *Z* = 1, *μ* = 3.629 mm^–1^, *ρ*_calcd_ = 1.677 Mg m^–3^, *R*_1_ = 0.0394, w*R*_2_ = 0.0793. The structure is a derivative of 1*, where uniaxial anisotropy has been deliberately introduced in the form of trigonal antiprismatic cobalt ions capped by the *fac*-tridentate ligand Tpm. The magnetic studies indicate that **1** exhibits long range antiferromagnetic ordering below 3.4 K and single chain magnetic behavior with Δ_eff_ = 39.7(3) cm^–1^ an astonishing enhancement compared to the properties of 1*.

## Experimental

### Starting materials

The ligand Tpm and [(*n*-Bu)_3_NH]_3_[W(CN)_8_] were prepared according to literature procedures.^21^ All other chemicals and solvents were of commercially available reagent grade quality and used as received.

### Physical measurements

Infrared (IR) spectra were measured as Nujol mulls placed between KBr plates on a Nicolet 740 FT-IR spectrophotometer. Direct current (dc) and alternating current (ac) susceptibility measurements were performed on a Quantum Design SQUID, Model MPMS XL-7 instrument. Single crystal X-ray data for **1** were collected on a Bruker APEX-II diffractometer equipped with CCD detectors at 110 K.

### Synthesis of {[(Tpm)Co(DMF)W(CN)_8_]_2_[Co(DMF)_4_]}_*n*_·2*n*DMF (**1**)

The reaction of CoCl_2_·6H_2_O (36.0 mg, 0.151 mmol) with [(*n*-Bu)_3_NH]_3_[W(CN)_8_]·4H_2_O (95.5 mg, 0.100 mmol) in 2 mL of DMF afforded a green solution which was filtered and then layered with 1 mL of DMF as a buffer solution followed by 2 mL of MeOH containing Tpm (30.3 mg, 0.141 mmol). Thin red platelet crystals were collected after two weeks. Yield: 47.5 mg, 48.0%. Anal. calcd C_60_H_76_Co_3_N_36_O_8_W_2_ (F.W. = 1974.06 g mol^–1^): C, 36.51; H, 3.88; N, 25.54. Found: C, 36.36; H, 3.98; N, 25.73. IR (Nujol, cm^–1^): 3489 (w, br), 3135 (m), 2197 (C≡N, w), 2176 (C≡N, w) and 2138 (C≡N, w) cm^–1^, 1651 (vs), 1519 (m), 1497 (m), 1456 (vs) 1404 (s), 1378 (vs), 1299 (m), 1288 (s), 1252 (s), 1221 (w), 1116 (m), 1093 (s), 1056 (s), 981 (m), 918 (w), 858 (s) (Fig. S1‡).

## Results and discussion

### Synthesis

Compound **1** was synthesized by first reacting CoCl_2_·6H_2_O in DMF with [(*n*-Bu)_3_NH]_3_[W(CN)_8_]·4H_2_O to form a green solution. After layering the reaction mixture with a solution containing the supporting ligand Tpm, crystals of **1** formed over the course of two weeks. An IR spectrum of the compound shows features at 2197 cm^–1^, 2176 cm^–1^, and 2138 cm^–1^, corresponding to the bridging and terminal cyanide stretches, respectively. Thermal gravimetric analysis (TGA) data revealed that the interstitial DMF solvent molecules (calcd 7.4%) are gradually lost before compound **1** begins to decompose at *ca.* 180 °C (Fig. S2‡). The phase purity of the product was verified by powder X-ray diffraction (PXRD) (Fig. S3‡).

### Structural description

**Fig. 1:**
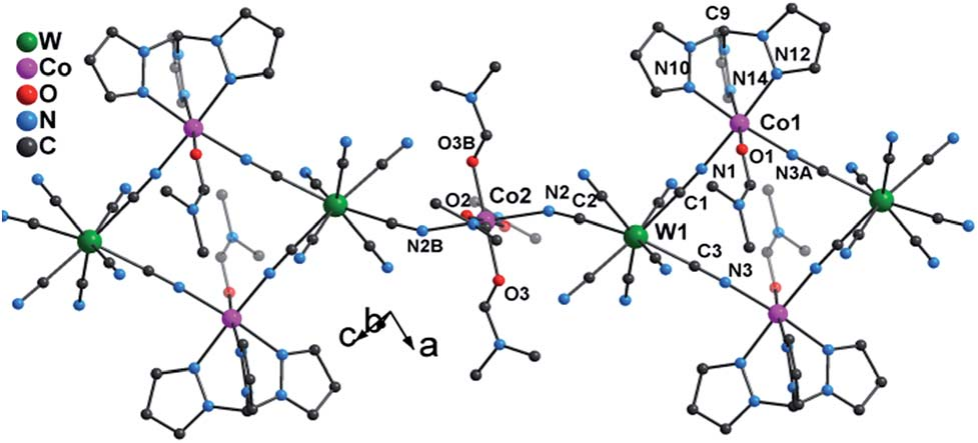
X-ray structure of **1**. All hydrogen atoms and lattice solvents are eliminated for the sake of clarity.

Single crystal X-ray diffraction studies revealed that **1** crystallizes in the triclinic *P*1 space group as a one-dimensional (1-D) array of [(Tpm)Co(DMF)W(CN)_8_]_2_^2–^ squares interconnected by [Co(DMF)_4_]^2+^ moieties, best described as a 3,2-chain^20^ (Fig. 1). The asymmetric unit of the structure consists of one [W(CN)_8_]^3–^ unit, and 1.5 Co^II^ centers bridged by cyanide. Each octacyanotungstate(v) unit (W1) is in a square antiprismatic geometry with an average W–C bond distance of 2.160(3) Å. The W1 ion is linked to three cobalt centers *via* cyanide groups. The remaining five cyanide ligands are terminal. The Co1 ion is coordinated to one DMF molecule and adopts a pseudo-octahedral geometry that is distorted to a trigonal anitiprismatic environment due to the presence of the capping ligand Tpm with intra-ligand bite angles of N–Co–N in the range of 82.69(1)–84.23(1)°. The other *cis* N–Co–N/O bond angles vary from 88.38(1)° to 97.33(1)°. The W1 and Co1 atoms are bridged by cyanide to form a [W_2_Co_2_]^2–^ square with a N–Co–N angle of 90.49(1)°.

The coordination geometries of Co1 in both **1** and 1* were compared to an ideal octahedron using the SHAPE program.^21^ The coordination geometry of Co1 in 1* is very close to an ideal octahedron (CShM = 0.111) while the coordination geometry of Co1 in **1** exhibits substantially more distortion from an ideal octahedron (CShM = 0.297). This distortion is similar to that observed for [Co(Tpm)_2_](BPh_4_)_2_ (CShM = 0.450) and is consistent with the assignment of the geometry of Co1 in **1** as trigonal antiprismatic rather than octahedral.

The Co2 ion is located on an inversion center and adopts an elongated octahedral geometry with four DMF molecules in the equatorial plane [Co–O = 2.043(2)–2.055(2) Å] and two [W^V^(CN)_8_]^3–^ units coordinated in *trans* apical positions [Co–N_cyanide_ = 2.139(2) Å]. The [W_2_Co_2_]^2–^ squares are further linked *via trans*-[Co^II^(DMF)_4_]^2+^ (Co2) fragments to form the overall 3,2-chain structure. The cyanide linkages within the square deviate slightly from linearity with W^V^–C–N and Co^II^–N–C bond angles of 177.35(2)–178.42(2)° and 172.67(2)–176.81(2)°, respectively. The cyanide linkages around Co2 are much more bent [Co2–N2–C2 = 155.9(5)° and W1–C2–N2 = 175.68(2)°], resulting in the nearest W–Co separation being 5.308(9) Å within the chain. The chains are well isolated with the closest inter-chain metal–metal distances being 7.908(8), 8.478(7), and 9.814(1) Å for Co···Co, Co···W and W···W, respectively; these values are comparable to those (8.21(2), 9.71(1), and 9.80(2) Å, respectively) found in the parent compound 1* (Fig. S4‡).

### Magnetic properties

**Fig. 2:**
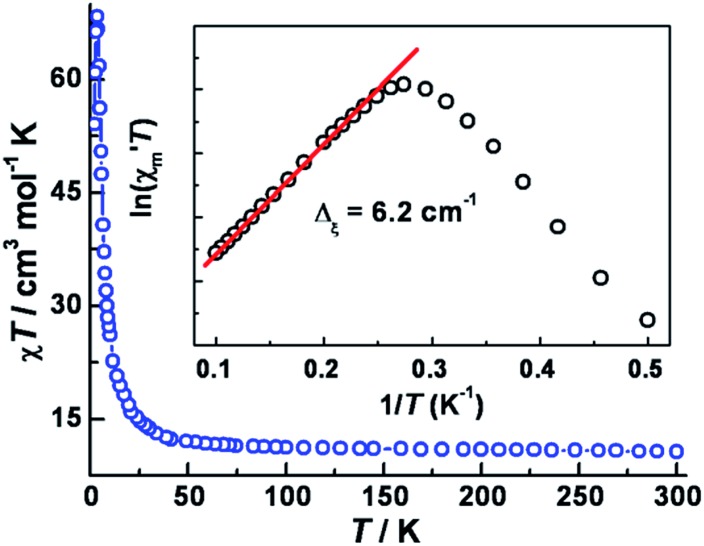
Variable-temperature dc magnetic susceptibility data of **1**, collected in an applied field of 1 kOe. The inset: plot of ln(*χ*′_m_*T*) *vs.* 1/*T* (where *χ*′_m_ is the molar component of the ac susceptibility at 1 Hz, *H*_ac_ = 5 Oe and *H*_dc_ = 0).

The variable temperature magnetic susceptibility data in an applied dc field of 1 kOe are plotted in Fig. 2. At 300 K, the *χ*_m_*T* value of 10.7 cm^3^ mol^–1^ K is much higher than the spin-only value (6.375 cm^3^ mol^–1^ K) for three Co^II^ (*S*_Co_ = 3/2, *g* = 2.0) and two W^V^ (*S*_W_ = 1/2, *g* = 2.0) centers, in accord with significant spin–orbital coupling with *g*_Co_ = 2.66. Upon lowering the temperature, the *χ*_m_*T* value remains nearly constant until ∼50 K and then increases abruptly to a maximum of 68.4 cm^3^ mol^–1^ K at 3.5 K, before decreasing to 51 cm^3^ mol^–1^ K at 2 K. These results indicate overall ferromagnetic coupling between W(v) and Co(ii) centers, consistent with other previously reported W^V^–Co^II^ complexes.^22^ No suitable model could be applied for further quantitative analysis of this system because of its complicated topology.

**Fig. 3:**
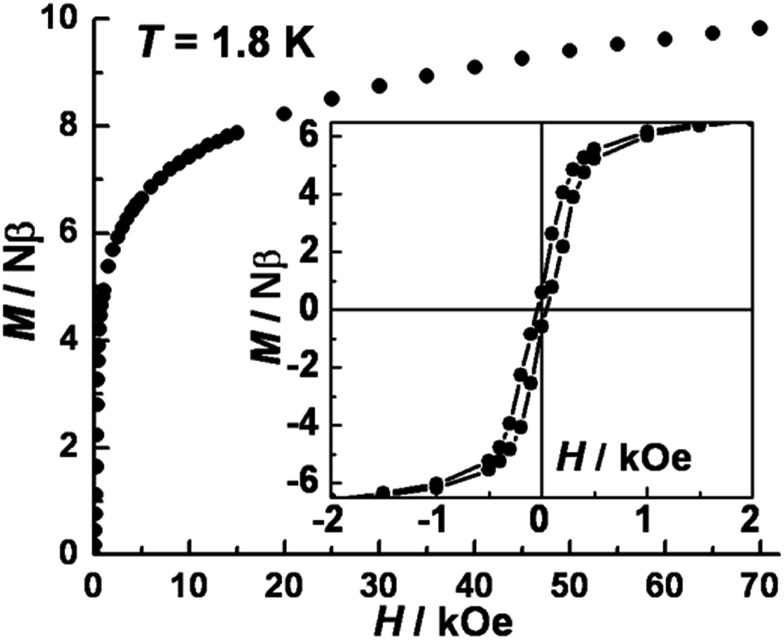
Plot of isothermal field-dependent magnetization at 1.8 K for **1**. Inset: magnetic hysteresis loop with a sweeping rate of 60 Oe per min at 1.8 K.

The isothermal field-dependent magnetization (*M vs. H*) was measured at temperatures ranging from 1.8–7.0 K (Fig. S5a‡). The magnetization at 1.8 K (Fig. 3) rapidly increases above zero field but does not saturate even at 7 T. The value of 9.82 Nβ at 7 T is much lower than the expected saturation value of ∼14.0 Nβ for three Co^II^ (*S*_Co_ = 3/2, *g* = 2.66) and two W^V^ (*S*_W_ = 1/2, *g* = 2.0) ions, due to significant magnetic anisotropy as corroborated by the non-superposition of the *M vs. H*/*T* plots at higher fields (Fig. S4b‡). Plots of d(*M*)/d(*H*) *vs. H* indicate a phase transition occurs from antiferromagnetic (AF) ordering to paramagnetic with a critical field of 300 Oe at 1.8 K (Fig. S6‡). A narrow hysteresis loop at 1.8 K was observed with a coercive field of ∼40 Oe and a remnant magnetization of 0.6 Nβ. zero-field-cooled (ZFC) and field-cooled (FC) magnetization data collected under a field of 20 Oe showed a sharp peak at 3.4 K and irreversibility below *ca.* 2.4 K; the former feature suggests an AF ordering while the latter behavior represents the blocking temperature (Fig. S6‡). Similar behavior has been noted for other cases of magnetic ordering in SCMs.^23^

In 1963, Glauber reported his prescient work describing an anisotropic Heisenberg or Ising-like one-dimensional system with the equation: *χ*_m_*T*/*C*_eff_ = exp(Δ_ξ_/*k*_B_*T*), where *C*_eff_ is the effective Curie constant, and *k*_B_ is the Boltzmann constant. From this relationship, *χ*_m_*T* is expected to increase exponentially with decreasing temperature under a zero applied dc field. Variable-temperature ac susceptibility data were collected in a zero applied dc field and an ac field of 5 Oe oscillating at 1 Hz (Fig. S7‡). The resulting plot of ln(*χ*′_m_*T*) *vs.* 1/*T* features a linear region in the temperature range 4.0–12 K, yielding Δ_ξ_ = 6.2 cm^–1^ (inset of Fig. 2). Below 4.0 K, ln(*χ*′_m_*T*) reaches a maximum and then undergoes a linear decrease with decreasing temperature.

**Fig. 4:**
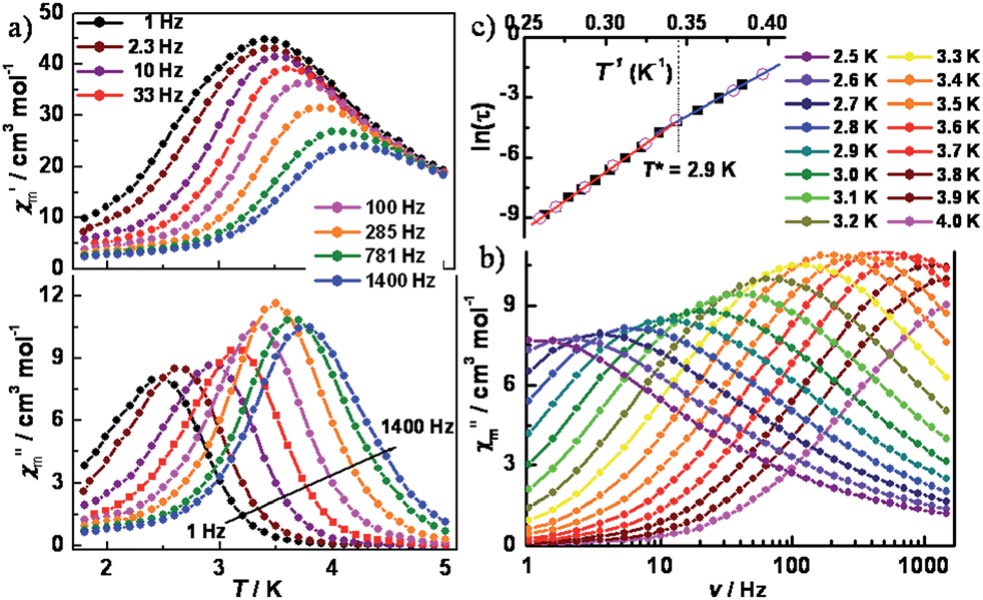
Variable-temperature (a) and variable-frequency (b) in-phase (*χ*′_m_) and out-of-phase (*χ*′′_m_) ac magnetic susceptibility data for **1**, collected in a 5 Oe ac field oscillating at temperatures of 1.8 to 5.0 K and frequencies of 1–1500 Hz. (c) Arrhenius plot of relaxation times, as determined through variable-temperature (○) and variable-frequency (■) ac susceptibility measurements.

To further probe the dynamics of the magnetization, ac magnetic susceptibility measurements were performed as a function of both temperature and frequency in a 5 Oe ac field and a zero dc field. As shown in Fig. 4a, variable-temperature ac susceptibilities for 1 display a strong frequency dependence of both in-phase (*χ*′_m_) and out-of-phase (*χ*′′_m_) components. The shift of the peak temperature (*T*_p_) of *χ*′_m_, as evaluated by the Mydosh parameter *φ* = (Δ*T*_p_/*T*_p_)/Δ(log *f*) ≈ 0.13, is consistent with normal superparamagnetic behavior (*φ* = 0.1–0.3).^24^ Variable-frequency ac susceptibilities collected in the range 2.5–4.0 K also show highly frequency dependent peaks (Fig. 4b and S8‡). The relaxation time, extracted from the peaks of *χ*′′_m_ in Fig. 4, follows two thermally activated laws, corresponding to infinite-size and finite-size regimes, respectively, marked by the crossover temperature of T* = 2.9 K (Fig. 4c). A fitting based on the Arrhenius relationship *τ* = *τ*_0_ exp(Δ/*k*_B_*T*) gave: Δ_1_ = 39.7(3) cm^–1^, *τ*_01_ = 3.4(5) × 10^–11^ s for the infinite regime and Δ_2_ = 31.8(2) cm^–1^, *τ*_02_ = 2.2(4) × 10^–9^ s for the finite regime. Given the relationship between the energy barriers for a SCM system, Δ_1_ = 2Δ_ξ_ + Δ_A_ and Δ_2_ = Δ_ξ_ + Δ_A_, the anisotropy (Δ_A_) and correlation energy (Δ_ξ_) were calculated to be 23.9 and 7.9 cm^–1^, respectively. The latter value is consistent with 6.2 cm^–1^ as estimated from the ln(*χ*′_m_*T*) *vs.* 1/*T* plot. The small correlation energy may be due to the weak magnetic coupling between the W(v) and Co(ii) ions across the cyanide bridge, which was found to be ∼0.7 cm^–1^, according to the 2*J* formalism of the exchange Hamiltonian.^22^ Based on |4Δ_A_/Δ_ξ_|, the |*D*/*J*| value was calculated to be 12.1, suggesting an Ising model for the current case. Cole–Cole plots (Fig. S9‡) of *χ*′′_m_*vs. χ*′_m_ were fit to a generalized Debye model,^25^ giving *α* values ranging from 0.24 to 0.50, indicative of a wide distribution of relaxation times, which could be caused by multiple relaxation processes, poly-dispersity of the chain length, magnetic interchain interactions, and/or random defects.

**Fig. 5:**
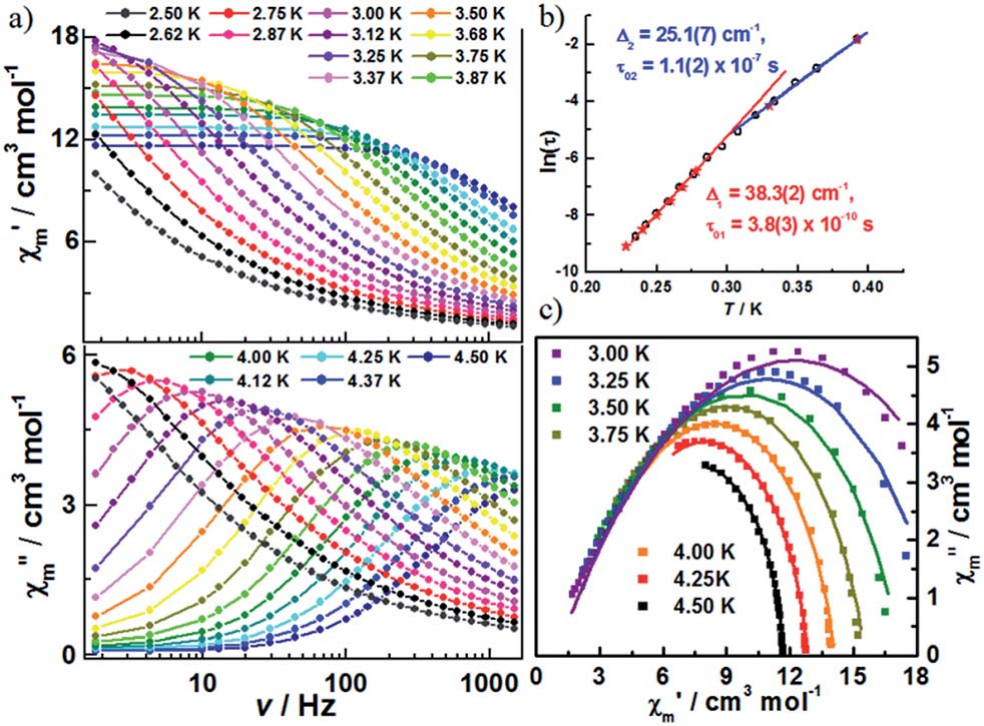
(a) Variable-frequency in-phase (*χ*′_m_) and out-of-phase (*χ*′′_m_) ac magnetic susceptibility data for **1**, collected in a 500 Oe dc field and 5 Oe ac field oscillating at temperatures of 2.5 to 4.5 K and frequencies of 1–1500 Hz; (b) Arrhenius plot of relaxation times, as determined from variable-temperature (★) and variable-frequency (○) ac susceptibility measurements; (c) selected Cole–Cole diagrams of **1** at 3.0–4.5 K; the solid lines are least-square fittings for a distribution of single relaxation processes with a generalized Debye model.

The decrease in the magnitude of the maximum intensity of *χ*′′_m_ upon lowering the temperature and frequencies is attributed to unaccounted for AF interchain interactions. To test this hypothesis, additional ac susceptibilities were measured under a dc field of 500 Oe, which minimizes the effect of antiferromagnetic interchain interactions (Fig. 5, S10 and S11‡). As shown in Fig. S11,‡ classical SCM behaviour is observed. The effective energy barrier for the infinite and finite regimes was estimated to be 38.3(2) and 25.1(7) cm^–1^, respectively (Fig. 5b). The difference between the two energy barriers is almost double that of Δ_ξ_, likely originating from the contribution of the applied dc field. The *α* values from fitting the Cole–Cole plot (Fig. 5c) based on a generalized Debye model were moderately reduced (in the range of 0.15–0.41), which suggests that the interchain interactions do not entirely account for the wide range of relaxation in **1**.

The rapidly increasing number of mononuclear SMMs in the literature indicates the progress that is being made in predicting magneto-structural correlations. Spin–orbit coupling, the main source of magnetic anisotropy, can be tuned with intentional structural modifications to target a specific geometry or distortion.8*a* Although achieving such correlations still remains a complex issue for octahedral Co(ii) systems, recent findings indicate that a majority of six-coordinate cobalt(ii) compounds, such as those in an elongated octahedral geometry, exhibit easy-plane anisotropy (*D* > 0).^26^ Very recently, both theory and experiment suggest that uniaxial magnetic anisotropy (*D* < 0) can be introduced and/or enhanced by increasing the axial trigonal distortion of the crystal field wherein the easy axis is coincident with the *C*_3_ axis. In 2013, the Gao group corroborated this principle with their report of a mixed-valence [Co_3_^III^Co^II^] cluster with a Co(ii) ion in a slightly distorted triangular prismatic geometry that exhibits a large uniaxial magnetic anisotropy (*D*_Co_ = –115 cm^–1^).^27^ In this vein, we recently demonstrated a successful way to realize very large negative *D* values on the order of –100 cm^–1^ by preparing trigonal antiprismatic mononuclear cobalt(ii) SMMs [Co(Tpm)_2_][X]_2_, complexes.^18^

**Fig. 6:**
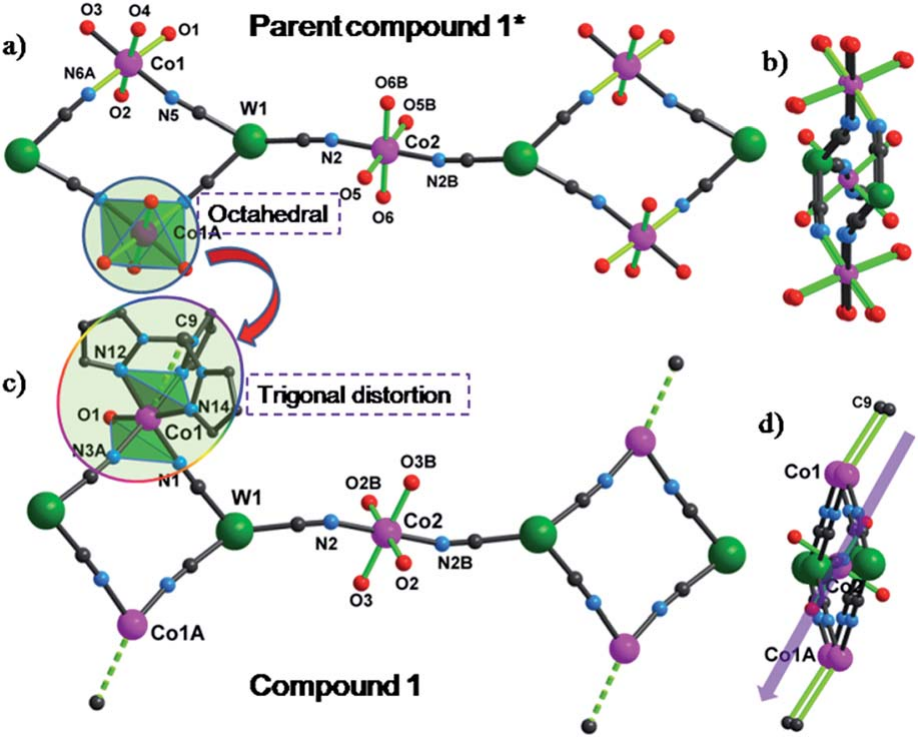
Truncated structure of the parent compound 1* (a) along and (b) through the chain direction; truncated structure of compound **1** (c) along and (d) through the chain direction. The green bonds represent easy axes of Co ions.

The SCM behaviour in **1** can be better understood by comparing the properties to the previously reported 1* in terms of their respective structures (Table S1‡). In 1*, both Co1 and Co2 centers adopt a slightly elongated octahedral geometry with a dihedral angle of 26.5° between their equatorial planes (easy-plane);^16^ whereas in **1**, while Co2 still adopts an elongated octahedral geometry (easy-plane anisotropy), the Co1 center is now trigonally distorted by virtue of the presence of the Tpm capping ligand which engenders uniaxial magnetic anisotropy along the *C*_3_ axis (Co1–C9). This very important distinction leads to differences in magnetic behaviour that can be rationalized by considering the easy axes of each compound, indicated by the green lines in Fig. 6. Compound 1* which is not an SCM does not have alignment of the easy plane anisotropy axes and is reported to exhibit glassy magnetic behavior and long range magnetic ordering. In the case of **1**, the Co1–C9 bond is nearly parallel to the Co2–O3 bond (one preferred orientation within the easy plane) with a small angle of 2.7°; therefore, the projection along the Co1–C9 direction of the anisotropy tensors results in Ising-like magnetic anisotropy with *g*_*z*_ > *g*_*x*_ ≠ *g*_*y*_, consistent with the above estimation (|*D*/*J*| > 4/3).

## Concluding remarks

This work highlights the advantage of incorporating the principles of mononuclear SMMs into the design of SCMs. The trigonal symmetry that is essential to the SMM properties of [Co(Tpm)_2_](BPh_4_)_2_ served as a guiding principle in the design of **1**. The use of Tpm in the synthesis of **1** engendered co-alignment of uniaxial magnetic anisotropies along the *C*_3_ axes of the Co^II^ moieties, resulting in good SCM behaviour, whereas its non-trigonal counterpart, 1*, is not an SCM. Future efforts to incorporate this trigonal distortion into SCMs with other cyanometallates as linkers will be explored.

## Conflicts of interest

There are no conflicts to declare.

## Supplementary Material

Supplementary information

Crystal structure data
