# Supplementary material for: Enforcing Ising-like magnetic anisotropy via trigonal distortion in the design of a W(v)–Co(ii) cyanide single-chain magnet
Source: Chem Sci. 2017 Nov 13;9(1):119–24. doi: 10.1039/c7sc02925j (PMC5873217; doi:10.1039/c7sc02925j)
Supplement: Supplementary file 1 [file SC-009-C7SC02925J-s001.pdf]

Electronic Supplementary Material (ESI) for Chemical Science.
This journal is © The Royal Society of Chemistry 2017

Supporting Information

Enforcing Ising-like magnetic anisotropy via trigonal distortion in the design of a W(V)-Co(II) cyanide single-chain magnet

Yuan-Zhu Zhang,[ab] Brian S. Dolinar,[a] Shihao Liu,[b] Andrew J Brown,[a] Xuan Zhang,[a] Zhao-Xi Wang,[a] and Kim R. Dunbar*,[a]

[a] Department of Chemistry, Texas A&M University, College Station, Texas 77842, USA. E-mail: dunbar@mail.chem.tamu.edu.

[b] Department of Chemistry, Southern University of Science and Technology, Shenzhen 518055, P.R. China.

Experimental Section

Materials and Physical Measurements.

All chemicals and solvents were used as received without further purification. The compounds 1,1,1-trispyrazoylmethane (Tpm) and $[(C_4H_9)_3NH]_3[W(CN)_8]$ ($[(C_4H_9)_3NH]$ = tri-n-butylammonium) were prepared according to the procedures in the literature. The IR spectra were recorded onpure samples on a Magna-IR 750 spectrophotometer in the 4000-500 cm^{-1} region. The measurements of variable–temperature magnetic susceptibility, ac magnetic susceptibility and field-dependence of the magnetization were performed on a Quantum Design MPMS-7 SQUID magnetometer. The experimental susceptibilities were corrected for the diamagnetism (Pascal's constants).[S1]

Single Crystal X-ray Diffraction Studies

Single crystal X-ray data for **1** were collected at 110 K on a Bruker APEX diffractometer equipped with a CCD detector. The data sets were recorded as ϖ-scans at 1.0° step widths. Integration was performed with the Bruker SAINT software package and absorption corrections were empirically applied using SADABS. The crystal structures were refined using the SHELX suite of programs. Images of the crystal structures were rendered using the visualization software DIAMOND. The structures were solved by direct methods and all non-hydrogen atoms were located by alternating cycles of least squares refinements and difference Fourier maps. All hydrogen atoms were placed at calculated positions except for some water molecules whose

hydrogen atoms were located by difference Fourier maps. The bond distances in disordered solvent molecules were restrained to chemically meaningful values. Anisotropic thermal parameters were added for all non-hydrogen atoms. A summary of pertinent information relating to unit cell parameters, data collection, and refinement statistics are provided in Table 1. CCDC 1508891 (**1**) contains the supplementary crystallographic data for this paper. These data can be obtained free of charge from the Cambridge Crystallographic Data Centre via www.ccdc.cam.ac.uk/data_request/cif.

[S1] G. A. Bain, J. F. Berry, *J. Chem. Edu.* **2008**, *85(4)*, 532-536.
[S2] *SMART and SAINT*, Siemens Analytical X-ray Instruments Inc., Madison, WI, USA, **1996.**
[S3] G. M. Sheldrick, *SADABS*, University of Gottingen, Gottingen, Germany, **1996**.
[S4] G. M. Sheldrick, *SHELXS-97 and SHELXL-97*, University of Gottingen, Gottingen, Germany, **1997.**
[S5] L. J. Barbour, *J. Supramol. Chem.* **2001,** *1*, 189–191

Table S1. Selected bond distances (Å) and angles (°) for **1**.

W1-C6	2.143(6)	N1-Co1-O1	90.52(17)
W1-C3	2.149(5)	N1-Co1-N3A	90.49(17)
W1-C8	2.159(6)	O1-Co1-N3A	91.44(16)
W1-C1	2.162(5)	N1-Co1-N12	175.35(18)
W1-C4	2.163(6)	N1-Co1-N14	91.52(17)
W1-C7	2.165(6)	N1-Co1-N10	94.58(17)
W1-C5	2.166(5)	N12-Co1-N10	84.23(16)
W1-C2	2.174(6)	N14-Co1-N10	82.69(17)
Co1-N1	2.061(5)	N12-Co1-N14	83.88(17)
Co1-O1	2.069(4)	O3B-Co2-N2	85.27(19)
Co1-N3A	2.085(4)	O3-Co2-N2	94.73(19)
Co1-N12	2.116(4)	O2-Co2-N2	90.21(16)
Co1-N14	2.129(5)	O2B-Co2-N2	89.79(16)
Co1-N10	2.135(4)	N2B-Co2-N2	180.000(1)
Co2-O3B	2.043(4)	N2-C2-W1	175.7(5)
Co2-O3	2.043(4)	N1-C1-W1	178.2(5)
Co2-O2	2.055(4)	N3-C3-W1	177.3(5)
Co2-O2B	2.055(4)	N4-C4-W1	177.9(5)
Co2-N2B	2.139(5)	N5-C5-W1	177.1(5)
Co2-N2	2.139(5)	N6-C6-W1	178.7(6)
C1-N1-Co1	176.8(4)	N7-C7-W1	179.2(5)
C2-N2-Co2	155.9(5)	N8-C8-W1	178.6(5)
C3-N3-Co1A	172.7(4)		
Symmetry transformations used to generate equivalent atoms: A -x,-y+1,-z+1; B -x+1,-y+1,-z			

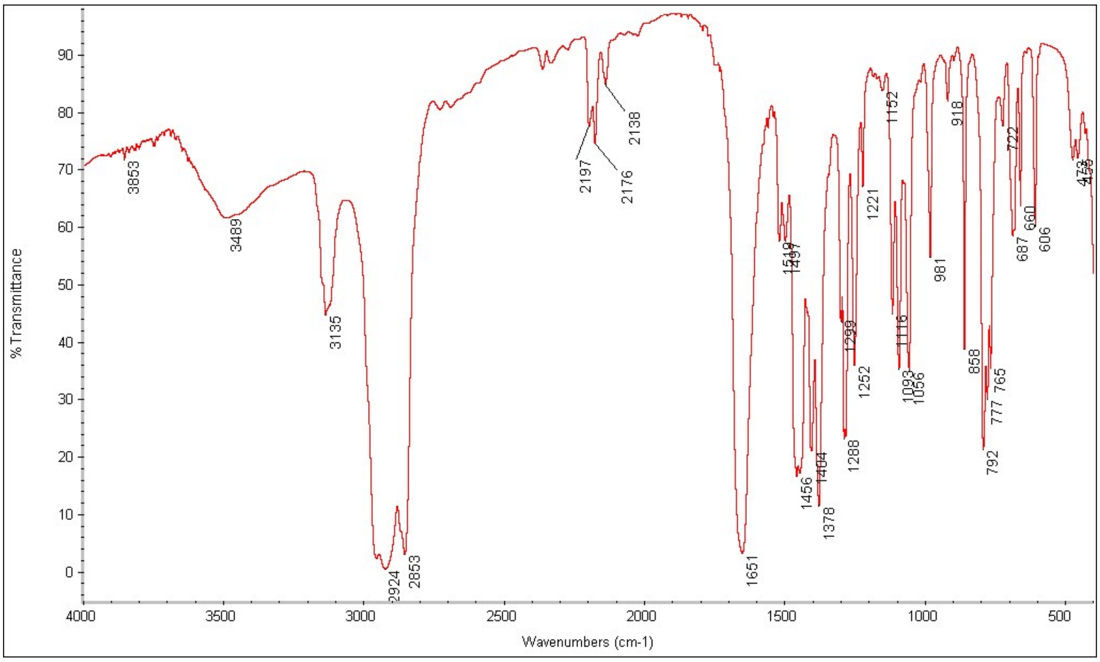

Fig. S1 The IR spectrum of **1** (nujol mull)

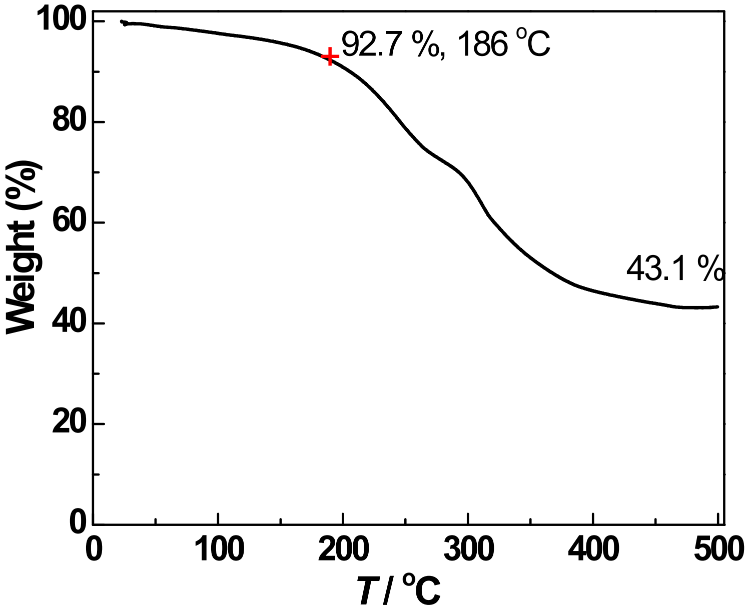

Fig. S2 TGA plot for **1**. (The DMF solvent (calcd. 7.4 %) is gradually lost before compound **1** starts to decompose at ca. 180 °C.)

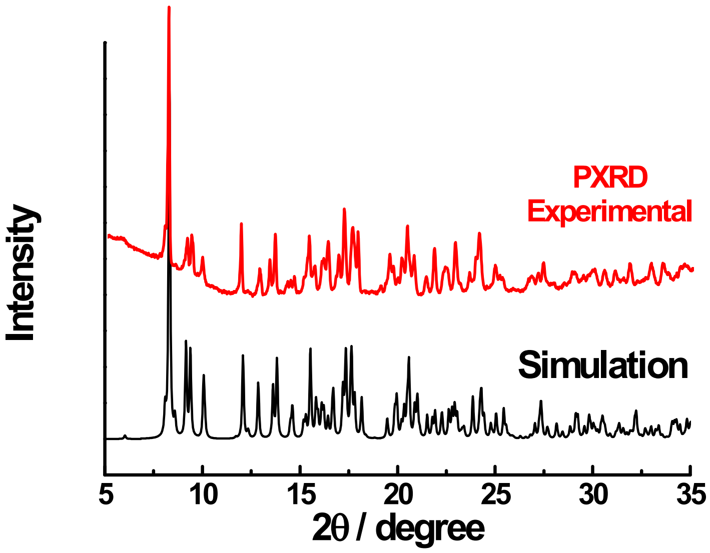

Fig. S3 Experimental and simulated powder x-ray diffraction patterns of **1**.

The powder X-ray diffraction (PXRD) data of ground fine powder were collected at room temperature on a sample **1** on a Rigaku SmartLab powder X-ray diffractometer with Cu Kα radiation (45 kV, 200 mA) between 5.0 and 35° (2θ) at ambient temperature. The PXRD data matches well with the calculated pattern from single crystal data.

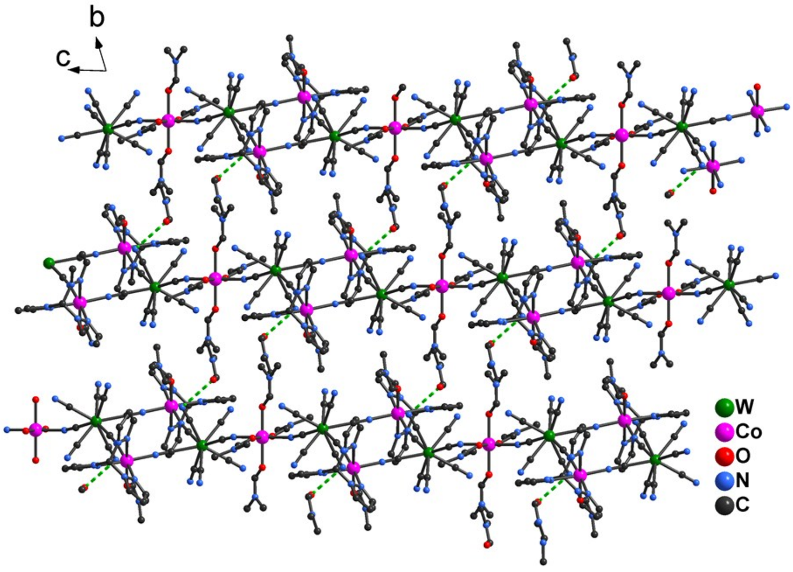

Fig.S4 Packing view of **1** showing a 3D structure with H-bonds between the chains and interstitial DMF solvents with $N_{cyanide}\cdots O_{DMF}$ distance of 3.226(6) Å.

(a)

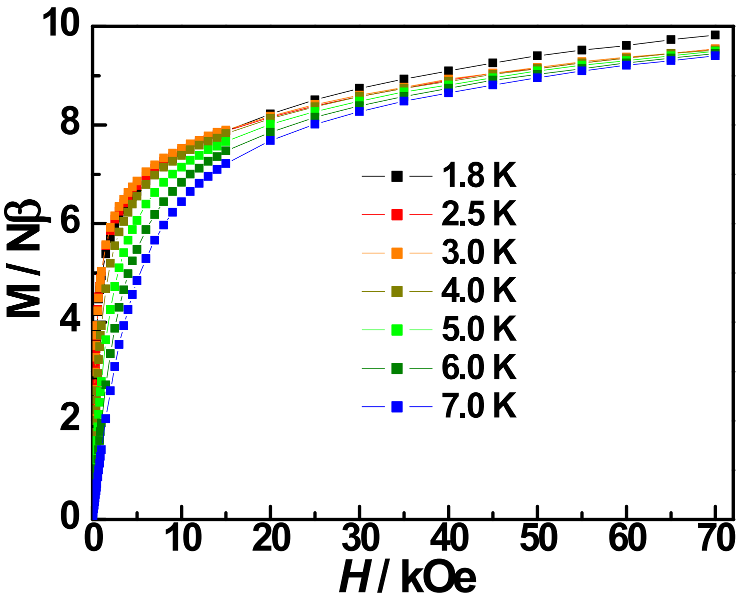

(b)

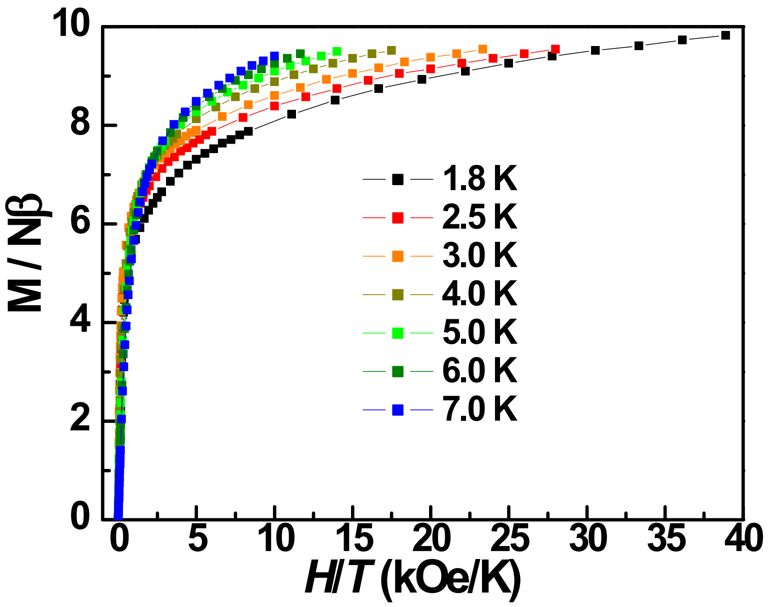

Fig. S5 (Top) Plots of isothermal field dependent magnetization for **1** at temperatures of 1.8 to 7.0 K; (Bottom) M vs. H/T plots at temperatures of 1.8 to 7.0 K for **1**. Solid lines are guide for eyes

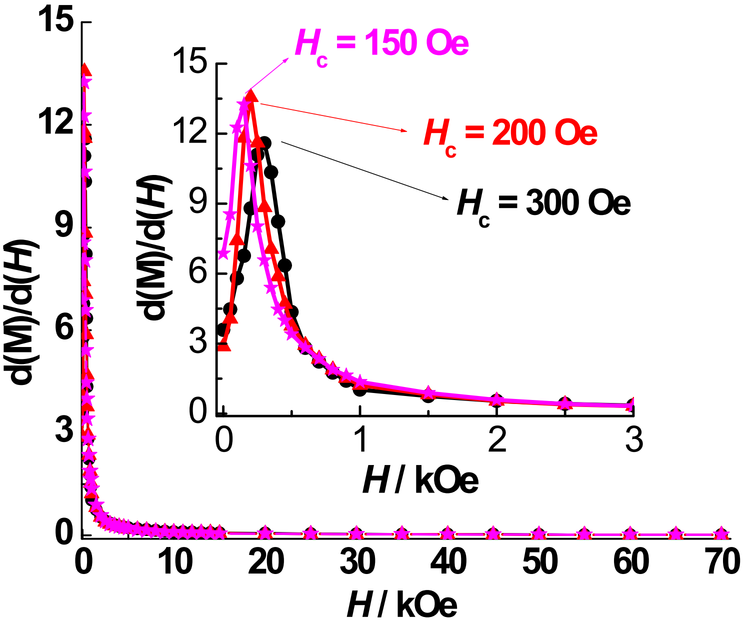

Fig. S6 d(M)/d(H) vs. H plots indicating a phase transition at H_c = 300, 200, and 150 Oe at temperatures of 1.8, 2.5 and 3.0 K, respectively.

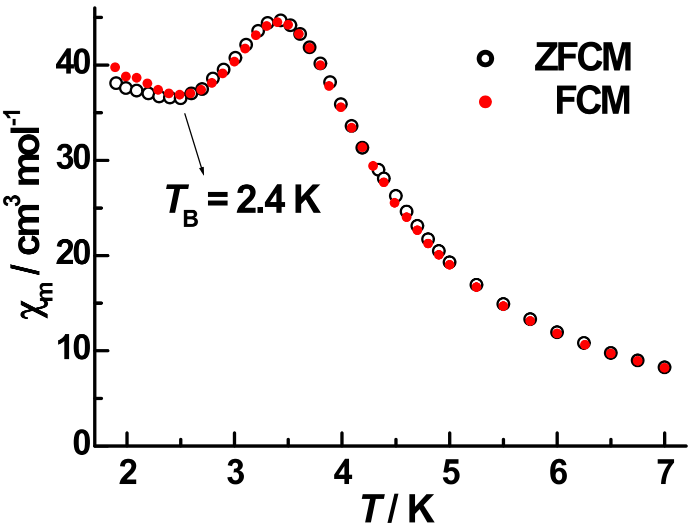

Fig. S7 Zero-field cooled (○) and field cooled (•) magnetization measured at 0.05 K/min at 20 Oe and at between 1.8 and 7.0 K.

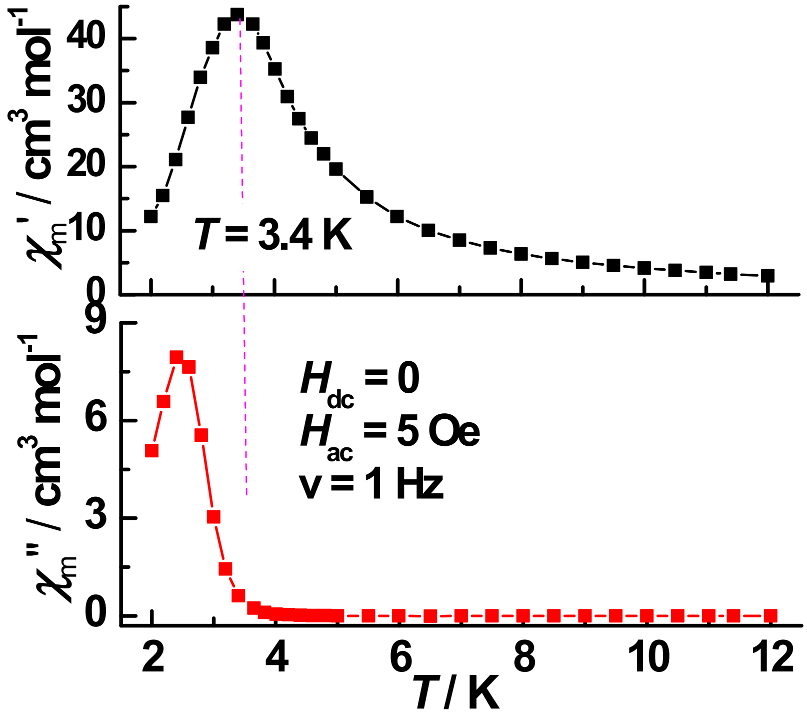

Fig. S8 Variable-temperature in-phase (χ_m', up) and out-of-phase (χ_m'', down) components of the ac magnetic susceptibility data for **1**, collected at temperatures of 2.0-12.0 K in a zero dc applied field and an ac field of 5 Oe.

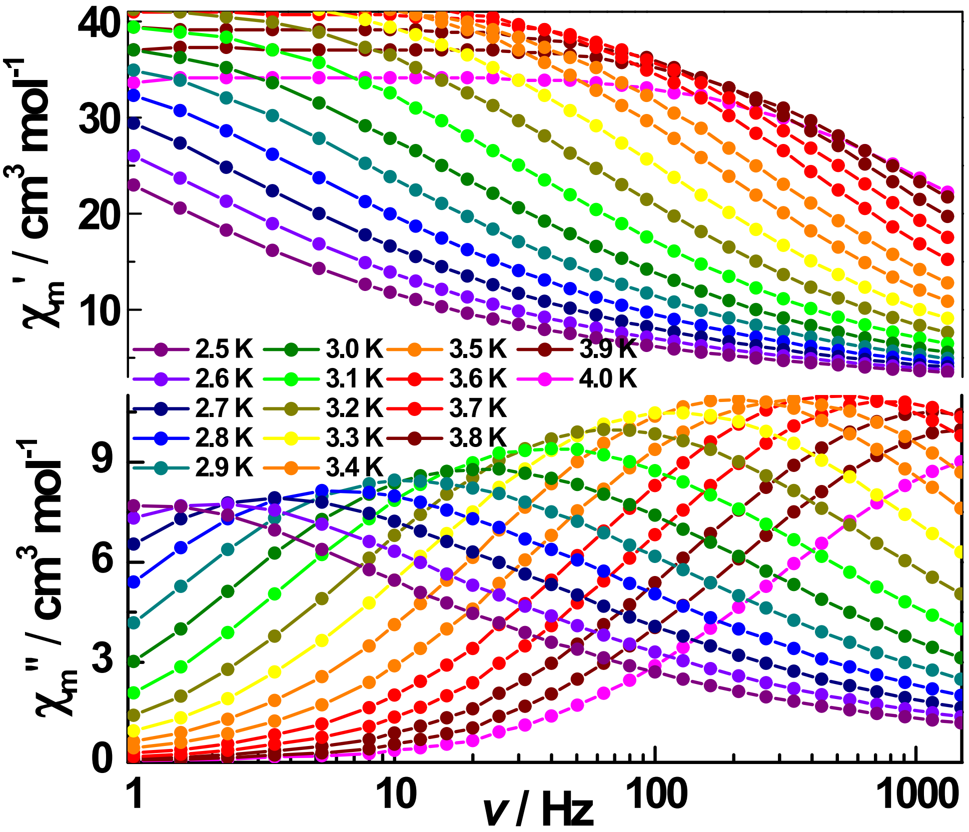

Fig. S9 Variable-temperature in-phase (χ_m', up) and out-of-phase (χ_m'', down) ac magnetic susceptibility data for **1**, collected in a 5 Oe ac field oscillating at temperatures of 1.8 to 5.0 K and frequencies of 1-1400 Hz.

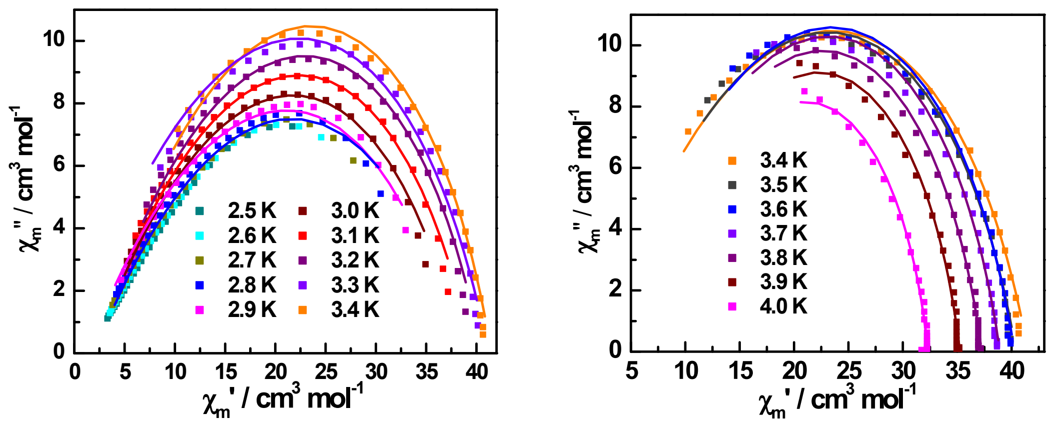

Fig. S10 Cole-Cole plots of **1** at 2.5 – 3.4 K (left) and 3.4-4.0 K in a zero dc field and ac field of 5 Oe. The solid lines are least-square fittings of the data to a distribution of single relaxation processes with a generalized Debye model.[a,b]

T / K	χ_s (cm^3 mol^{-1})	χ_t (cm^3 mol^{-1})	τ (S)	α	R
2.8	2.02528	38.505100	0.02531	0.5027788	4.92×10^{-4}
2.9	1.25992	38.00018	0.01100	0.49017	7.61×10^{-4}
3.0	1.99824	38.44664	0.00684	0.45830	4.56×10^{-4}
3.1	2.01117	39.52891	0.00382	0.43598	4.28×10^{-4}
3.2	1.99146	40.51225	0.00222	0.41553	3.56×10^{-4}
3.3	0.00049	41.17551	0.00110	0.42007	5.16×10^{-4}
3.4	2.20886	41.49723	0.00083	0.37608	2.49×10^{-4}
3.5	2.57618	40.39595	0.00054	0.35800	2.14×10^{-4}
3.6	3.37398	40.25919	0.00037	0.33621	1.69×10^{-4}
3.7	4.37882	38.92354	0.00026	0.31550	1.50×10^{-4}
3.8	5.33471	37.25133	0.00019	0.29689	1.10×10^{-4}
3.9	7.14904	35.24412	0.00015	0.26672	8.95×10^{-5}
4.0	8.12117	32.32569	0.00012	0.24493	7.29×10^{-5}

Refs: (a) Cole, K. S.; Cole, R. H. *J. Chem. Phys.* **1941**, 9, 341. (b) Aubin, S. M.; Sun, Z.; Pardi, L.; Krzysteck, J.; Folting, K.; Brunel, L.-J.; Rheingold, A. L.; Christou, G.; Hendrickson, D. N. *Inorg. Chem.* **1999**, 38, 5329.

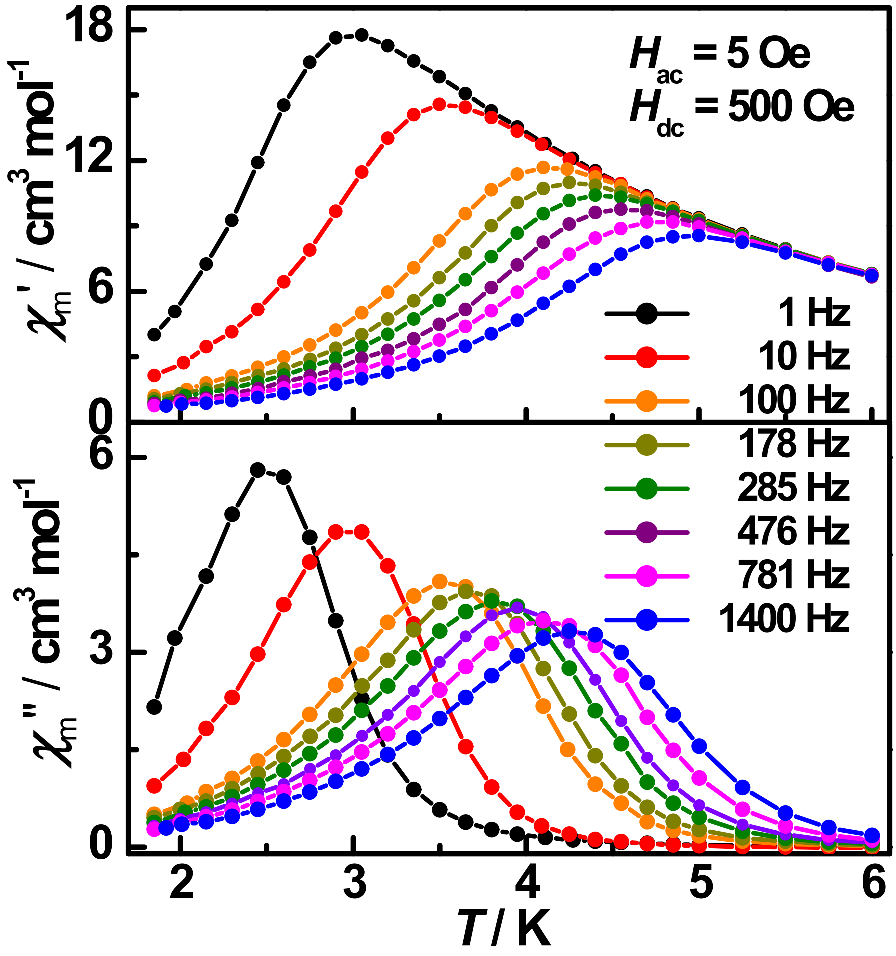

Fig. S11 Variable-temperature in-phase (χ_m', top) and out-of-phase (χ_m'', bottom) components of the ac magnetic susceptibility data for **1** collected at between 2.0-12.0 K with a 500 Oe dc applied field and an ac field of 5 Oe.

Table S1 The least-square fittings of the Cole-Cole data for **1** at 3.0 – 4.5 K with a 500 dc field and an ac field of 5 Oe to a distribution of single relaxation processes with a generalized Debye model.

T / K	χ_s (cm^3 mol^{-1})	χ_t (cm^3 mol^{-1})	τ (S)	α	R
3.00	0.92321	21.74473	0.01969	0.41921	7.27×10^{-4}
3.25	1.03071	19.26482	0.00602	0.38539	6.84×10^{-4}
3.50	1.17335	17.23045	0.00203	0.34701	6.00×10^{-4}
3.75	1.45042	15.55454	0.00081	0.30227	4.12×10^{-4}
4.00	1.81741	14.11411	0.00035	0.26343	2.75×10^{-4}
4.25	2.46140	12.83900	0.00018	0.21114	1.13×10^{-4}
4.50	3.34548	11.70651	0.00010	0.15266	3.11×10^{-5}

Table S2. Seleceted bond distances (Å) and angles (°) for **1*** and **1**.

**1***		**1**	
Co1-N5	2.131(3)	Co1-N1	2.061(5)
Co1-N6A	2.109(3)	Co1-N3A	2.085(4)
Co1-O2	2.083(3)	Co1-O1	2.069(4)
Co1-O3	2.097(3)	Co1-N12	2.116(4)
Co1-O1	2.111(3)	Co1-N10	2.135(4)
Co1-O4	2.068(3)	Co1-N14	2.129(5)
Co2-O5	2.070(4)	Co2-O2	2.055(4)
Co2-O6	2.084(3)	Co2-O3	2.043(4)
Co2-N2	2.096(3)	Co2-N2	2.139(5)
O3-Co1-O1	92.03(12)	N12-Co1-N10	84.23(16)
O4-Co1-O1	88.04(12)	N14-Co1-N10	82.69(17)
O3-Co1-O4	89.35(12)	N12-Co1-N14	83.88(17)
N5-Co1-N6A	93.78(13)	N1-Co1-N3A	90.49(17)
N5-Co1-O2	89.50(13)	N1-Co1-O1	90.52(17)
O2-Co1-N6A	88.52(12)	O1-Co1-N3A	91.44(16)
N2-Co2-O5	91.51(14)	N2-Co2-O3	90.21(16)
N2-Co2-O6	93.04(13)	N2-Co2-O2	94.73(19)
N2-Co2-O5B	88.50(13)	N2-Co2-O2B	89.79(16)
N2-Co2-O6B	86.96(14)	N2-Co2-O3B	85.27(19)
N2-Co2-N2B	180.00	N2-Co2-N2B	180.00
Symmetry transformations used to generate equivalent atoms: For **1***, A –x+1,-y+2,-z+1; B -x+2,-y+1,-z; For **1**, A -x,-y+1,-z+1; B -x+1,-y+1,-z;			
